# Effects of a 12-Week Zumba Training Program on Estimated Aerobic Capacity and Exploratory Hematological and Inflammatory Outcomes in Overweight/Obese Postmenopausal Women: A Pilot Randomized Controlled Trial

**DOI:** 10.3390/sports14100441

**Published:** 2026-10-08

**Authors:** Hanen Saadouni, Hamza Marzouki, Nejmeddine Ouerghi, Okba Selmi, Soukeina Hattabi, Vlad Adrian Geantă, Viorel Petru Ardelean, Vasile Emil Ursu, Daniela Giconda Burac, Jakob L. Vingren, Anissa Bouassida

**Affiliations:** 1 High Institute of Sports and Physical Education of Kef, University of Jendouba, Boulifa University Campus, Kef 7100, Tunisia; hanen.saadouni12@gmail.com (H.S.); hamzic_30@hotmail.com (H.M.); najm_ouerghi@hotmail.com (N.O.); okbaselmii@yahoo.fr (O.S.); hattabisoukaina@gmail.com (S.H.); bouassida_anissa@yahoo.fr (A.B.); 2 Research Unit “Sport Sciences, Health and Movement” (UR22JS01), University of Jendouba, Kef 7100, Tunisia; 3 Department of Physical Education and Sport, Faculty of Physical Education and Sport, Aurel Vlaicu University of Arad, 310025 Arad, Romania; viorel.ardelean@uav.ro; 4 Department of Physical Education and Sport, Faculty of Law and Social Sciences, University “1 Decembrie 1918” of Alba Iulia, 510009 Alba Iulia, Romania; vasile.ursu@uab.ro; 5 Department of Physical Education and Sport, Stefan cel Mare University, 720229 Suceava, Romania; 6 Department of Kinesiology, Health Promotion and Recreation, University of North Texas, Denton, TX 76203, USA; jakob.vingren@unt.edu

**Keywords:** menopause, cardiorespiratory fitness, blood indices, leukocyte profile, inflammation, exercise intervention

## Abstract

This pilot randomized controlled trial investigated the effects of a 12-week Zumba training program on estimated aerobic capacity in overweight/obese postmenopausal women, while hematological and inflammatory outcomes were explored. Thirty overweight/obese postmenopausal women were randomly assigned to a Zumba group (ZG, *n* = 15) or a control group (CG, *n* = 15). Eight participants withdrew, and 22 completers (11/group) completed the study and were included in the per-protocol analysis. The ZG completed supervised 60 min sessions three times weekly for 12 weeks, whereas the CG maintained its usual lifestyle habits. Estimated maximal oxygen uptake (VO_2_max), maximal aerobic speed (MAS), hematological parameters, and inflammatory biomarkers were assessed before and after the intervention. Intervention effects were assessed using baseline-adjusted analysis of covariance (ANCOVA). Estimated VO_2_max was higher in the ZG than CG at follow-up (adjusted difference: 3.59 mL·kg^−1^·min^−1^; partial eta squared (ηp^2^) = 0.683). Exploratory differences were observed for mean platelet volume (0.701 fL; ηp^2^ = 0.400) and neutrophils (0.961 × 10^3^/μL; ηp^2^ = 0.287), whereas no clear effects were identified for other hematological outcomes. Neither C-reactive protein nor erythrocyte sedimentation rate showed a clear effect. Zumba may improve estimated aerobic capacity, while the hematological findings remain exploratory and hypothesis-generating.

## 1. Introduction

Menopause is characterized by the permanent cessation of menstruation and a marked decline in ovarian estrogen production. Beyond reproductive aging, the menopausal transition is accompanied by unfavorable physiological changes, including increased visceral adiposity, impaired vascular function, reduced insulin sensitivity, and a higher cardiovascular risk profile than would be expected from chronological aging alone [1,2]. In parallel, postmenopausal women frequently exhibit lower cardiorespiratory fitness, an important concern because reduced cardiorespiratory fitness is strongly associated with higher risks of all-cause and cardiovascular mortality [3]. These alterations may be especially pronounced in women with overweight or obesity, in whom excess adiposity is commonly linked to low-grade systemic inflammation and altered hematologic-inflammatory status [4]. These changes highlight the need for effective non-pharmacological strategies capable of improving both aerobic fitness and broader physiological health in this population.

Regular exercise is a cornerstone non-pharmacological strategy for improving health after menopause. Recent evidence indicates that structured exercise training effectively enhances cardiorespiratory fitness and muscular strength in postmenopausal women, with beneficial effects observed after aerobic, resistance, and combined training programs [5]. Aerobic exercise may also improve cardiometabolic health outcomes in postmenopausal females, although the magnitude of adaptation appears to depend on exercise prescription and participant characteristics [6]. Similarly, group aerobic exercise has been shown to improve body composition and lipid profile in young women with elevated body mass index (BMI) [7]. However, despite these benefits, adherence to conventional exercise programs is often suboptimal in previously inactive middle-aged women. This limitation underscores the importance of identifying exercise modalities that are not only physiologically effective but also enjoyable and sustainable over time.

Zumba is a dance-based fitness modality that combines rhythmic whole-body movement, music, and intermittent moderate-to-vigorous effort in a group setting. Its popularity is partly explained by its enjoyable and socially engaging format, which may favor long-term adherence [8]. A systematic review by Vendramin et al. [8] concluded that Zumba may be effective for improving aerobic capacity and selected health-related outcomes. More recent evidence has shown that an 8-week Zumba program can improve respiratory function in healthy inactive women, supporting its potential as an accessible aerobic training strategy [9]. Accordingly, Zumba may represent a promising alternative to more conventional exercise formats for postmenopausal women, particularly those with overweight or obesity. However, evidence remains limited in this specific population, especially when aerobic outcomes are examined together with blood-based physiological markers.

In addition to improvements in aerobic capacity and cardiometabolic health, hematological and inflammatory markers may provide useful insight into the systemic effects of exercise. Complete blood count-derived indices and related hematological parameters are clinically accessible and may reflect inflammatory, immune, and hematological status. Obesity has been associated with altered hematologic inflammatory markers, suggesting that routine blood indices may help characterize obesity-related systemic dysregulation [4]. In postmenopausal women with overweight or obesity, a recent systematic review and meta-analysis showed that exercise (i.e., aerobic, resistance, and combined training) may reduce pro-inflammatory markers and improve adiponectin levels, although effects vary by exercise modality and marker, with aerobic exercise appearing to produce the most consistent anti-inflammatory response [10].

However, despite extensive evidence showing that exercise training can improve cardiorespiratory fitness in postmenopausal women, considerably less is known about whether alternative and potentially more enjoyable exercise modalities such as Zumba produce comparable benefits in overweight/obese postmenopausal women. Furthermore, studies simultaneously examining aerobic capacity, hematological variables, and inflammatory biomarkers in this population are scarce. Consequently, the present study was designed not simply to evaluate whether exercise improves aerobic fitness, but to determine whether a structured Zumba intervention can elicit aerobic and physiological adaptations in an understudied population using a modality that may promote long-term adherence. Therefore, the aim of the present study was to investigate whether a 12-week Zumba training program, an exercise modality that may offer advantages for enjoyment and adherence, could improve aerobic capacity and influence hematological/inflammatory markers in overweight/obese postmenopausal women. We hypothesized that Zumba training would be associated with an improvement in estimated aerobic capacity, reflected by an increase in estimated maximal oxygen consumption (VO_2_max), and may induce favorable changes in selected hematological and inflammatory markers, particularly neutrophils (NEs), monocytes (MOs), mean platelet volume (MPV), C-reactive protein (CRP), and erythrocyte sedimentation rate (ESR), compared with a non-exercising control group.

## 2. Materials and Methods

### 2.1. Participants

Prior to recruitment, an a priori sample-size calculation was conducted using G*Power (version 3.1.9.4; University of Kiel, Kiel, Germany) for a repeated-measures ANOVA assessing the within-between interaction (group × time) [11]. The calculation was informed by effect-size assumptions derived from a comparable exercise trial [12] and used a conservative interaction effect size of f = 0.40, with α = 0.05 and statistical power = 0.80. Under these assumptions, the minimum required sample was 16 participants in total (8 per group). Because the sample-size calculation was not based on each individual aerobic, hematological, and inflammatory endpoint reported in the present manuscript, caution is warranted when interpreting outcomes that are known to show greater biological variability, particularly the hematological and inflammatory markers. The sample-size calculation was not designed to provide separate statistical power for each individual endpoint examined in the present secondary analysis; therefore, the hematological and inflammatory outcomes should be considered exploratory. Potential participants were recruited through flyers, advertisements, and local announcements distributed in community centers, local associations, primary care clinics, and academic establishments. After screening and baseline assessment, 30 overweight or obese postmenopausal women aged 44–60 years met the eligibility criteria and were enrolled in the study. Participants were randomly assigned in a 1:1 ratio to either a Zumba training group (ZG; *n* = 15) or a non-exercising control group (CG; *n* = 15). Randomization was performed using permuted blocks and stratified according to baseline body mass index (BMI) to ensure balanced group allocation. To minimize selection bias, eligibility determination and baseline assessments were performed by an assessor not involved in the randomization procedure, and allocation was disclosed only after eligibility confirmation and completion of baseline testing.

During the intervention, eight participants withdrew for personal reasons (ZG, *n* = 4; CG, *n* = 4). Although baseline assessments were conducted before randomization in the original trial, the individual-level baseline raw data for the eight participants who subsequently withdrew could not be retrieved from the archived study dataset available for the present secondary analysis. Consequently, baseline characteristics of completers and non-completers could not be compared, limiting our ability to directly assess potential attrition bias. No adverse events were reported during the intervention period. According to the predefined adherence criteria, participants in the ZG were required to attend at least 90% of the scheduled training sessions, and all participants were required to complete the pre- and post-intervention assessments to be included in the final analysis. The present study therefore reports per-protocol effects among study completers. Because the individual-level data required for analysis could not be retrieved for participants who withdrew, intention-to-treat or modified intention-to-treat analyses could not be performed.

**Figure 1:**
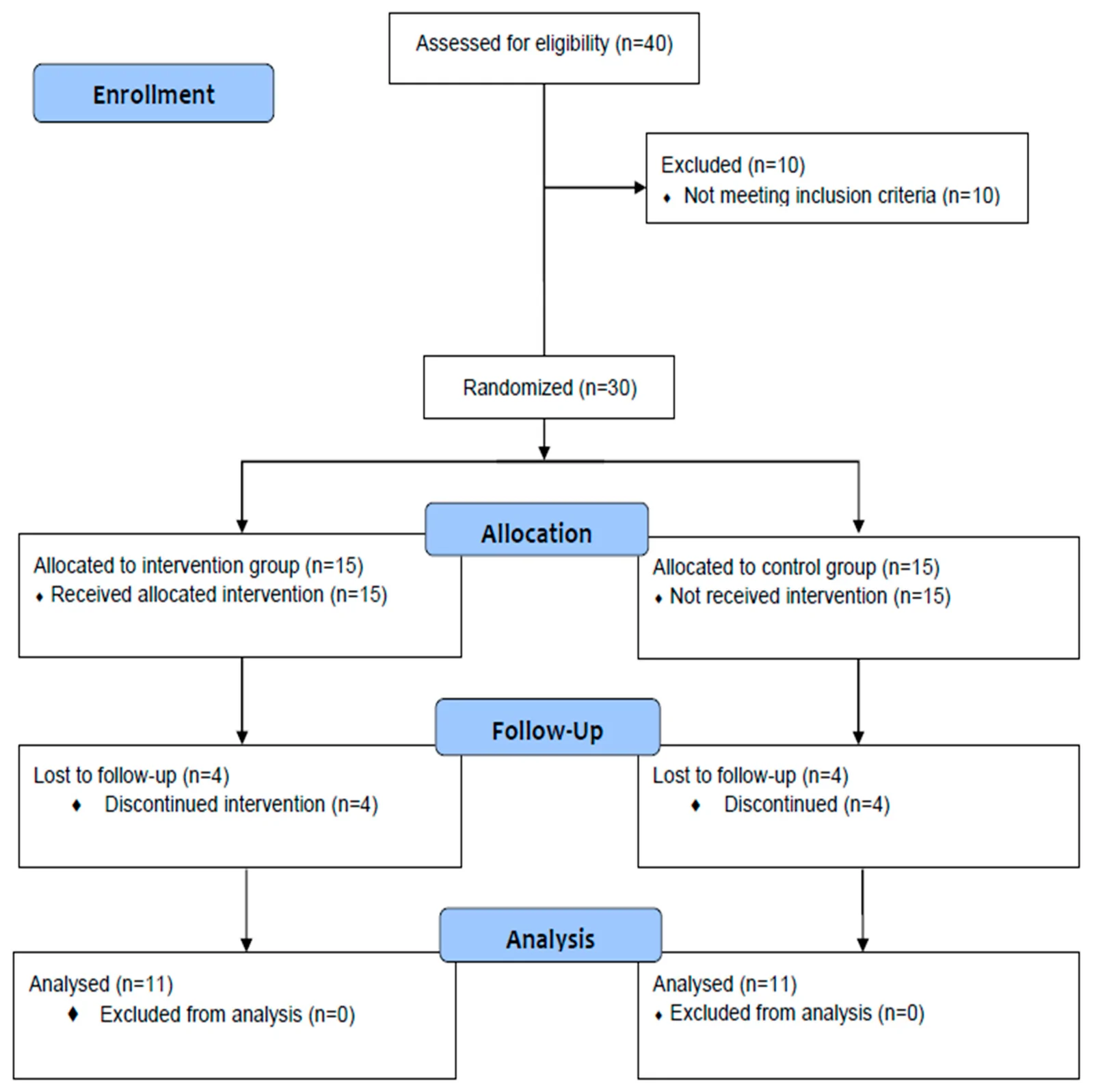
CONSORT flow diagram.

Accordingly, 22 participants completed the study and were included in the per-protocol analysis: ZG (*n* = 11; age: 52.6 ± 4.31 years; height: 161.6 ± 4.5 cm; body mass: 80.0 ± 16.1 kg; BMI: 30.9 ± 5.4 kg·m^−2^; body fat percentage [%BF]: 37.1 ± 2.9%) and CG (*n* = 11; age: 55.7 ± 4.33 years; height: 160.4 ± 4.8 cm; body mass: 83.0 ± 13.2 kg; BMI: 31.8 ± 5.0 kg·m^−2^; %BF: 37.9 ± 4.8%). Participant flow, exclusions, and reasons for withdrawal are presented in Figure 1 (CONSORT flow diagram).

Inclusion criteria were age 44–60 years; BMI ≥ 25 kg·m^−2^; postmenopausal status based on clinical history (≥12 consecutive months of amenorrhea) with ≥3 years since the last menstrual period; and stable resting heart rate. Exclusion criteria included ongoing therapy for obesity, chronic illness, endocrine disorders, diabetes mellitus, or any contraindication to exercise training. All participants underwent a medical evaluation prior to enrollment (clinical history and resting heart rate assessment). Women with cardiovascular, metabolic, thyroid, or inflammatory diseases, or those receiving medications likely to influence metabolic or hormonal outcomes, were excluded. During the intervention, participants were instructed to maintain their usual dietary habits; dietary intake was not formally monitored. All participants provided written informed consent before enrollment. The study was conducted in accordance with the Declaration of Helsinki and approved by the local institutional ethics committee (Approval Number: ISSEPK/12-2023).

### 2.2. Experimental Protocol

This study aimed to assess the effects of a 12-week Zumba training program on various physiological and biological parameters in sedentary and overweight or obese postmenopausal women. The ZG engaged in three supervised training sessions per week, each lasting approximately 60 min, for the 12-week intervention period. The CG did not participate in any structured physical activity. Before and after the 12-week intervention period, aerobic capacity (estimated VO_2_max and maximal aerobic speed [MAS]), hematological parameters (white blood cells [WBCs], red blood cells [RBCs], hemoglobin [HB], hematocrit, mean corpuscular volume [MCV], mean corpuscular hemoglobin [MCH], mean corpuscular hemoglobin concentration [MCHC], platelets [PLTs], mean platelet volume [MPV], neutrophils [NEs], lymphocytes [LYs], monocytes [MOs], eosinophils [EOs], and basophils [BAs]), and inflammatory biomarkers (C-reactive protein [CRP] and erythrocyte sedimentation rate [ESR]) were measured. The present study represents a secondary analysis of the same randomized cohort and 12-week Zumba intervention previously reported in [13]. The previous publication focused on thyroid and cardiometabolic outcomes, whereas the present analysis focuses on estimated aerobic capacity, hematological parameters, and systemic inflammatory biomarkers. Outcomes and analyses previously reported in [13] are not reproduced in the present manuscript.

To minimize the impact of environmental and circadian factors on performance, all physical tests and training sessions were conducted on the same outdoor field and at the same time of day (5:00 p.m. to 6:00 p.m.). This time window was selected to ensure stable weather conditions, avoid the influence of extreme temperatures, and reduce variability in performance due to environmental conditions. Participants were instructed to avoid any intense physical activity for at least 48 h prior to testing and to refrain from eating for at least three hours before each session. No dietary interventions were implemented during the study, and participants were advised to continue following their normal dietary habits throughout the research period. Participants were also instructed to maintain their usual physical activity and other lifestyle behaviors throughout the study period. The study was conducted from June to September 2023.

### 2.3. Morphological Measurements

To reduce measurement bias, all morphological assessments were performed by the same trained evaluator using identical equipment under standardized conditions. Participants were assessed barefoot and wearing light clothing. Stature was measured to the nearest 0.1 cm using a Harpenden stadiometer (Holtain Instruments, Crosswell, Crymych, Pembrokeshire, UK), and body mass was recorded to the nearest 0.1 kg using an electronic scale (Tanita, Model TBF-410 GS, Tokyo, Japan). BMI was calculated as body mass divided by height squared (kg·m^−2^).

Subcutaneous adiposity was estimated from skinfold thickness measured at four standard anatomical sites on the right side of the body (biceps, triceps, subscapular, and suprailiac) using a Harpenden caliper (Holtain Instruments, Crosswell, Pembrokeshire, UK). Each skinfold was measured three times, and the recorded values were used to estimate body density according to a validated method for adult women [14]. Body fat percentage was then calculated using Siri’s equation [15].

### 2.4. Aerobic Capacity Assessment

Assessment of estimated VO_2_max and MAS was performed using a progressive running test to exhaustion based on the VAMEVAL protocol developed by Cazorla and Léger [16]. Briefly, the test was conducted on a 200 m athletics track, during which participants followed a progressively increasing pace guided by audio signals. Markers were placed every 20 m using cones to help participants adjust their running speed. The test began at a speed of 8 km·h^−1^, which increased by 0.5 km·h^−1^ every minute until participants were no longer able to maintain the required pace. The test was terminated when a participant failed to reach the designated marker in time for two consecutive audio signals or voluntarily stopped because of exhaustion. This protocol was selected because it allows estimation of aerobic capacity through MAS and derived VO_2_max, which matched the aerobic outcomes of interest in the present study. It was considered more appropriate than functional field tests that mainly assess general exercise capacity but do not directly provide MAS or a field-test-derived estimate of VO_2_max. Although the VAMEVAL protocol has not been specifically validated in overweight/obese postmenopausal women, its selection was supported by previous studies that have used this protocol to assess maximal aerobic speed and estimate VO_2_max in overweight/obese populations. MAS was defined as the speed attained during the last fully completed stage. VO_2_max was then estimated from MAS using the following equation: estimated VO_2_max (mL·kg^−1^·min^−1^) = MAS (km·h^−1^) × 3.5.

### 2.5. Training Program

The ZG participated in a structured 12-week Zumba exercise program performed three times per week (Monday, Wednesday, and Friday) under the supervision of a certified Zumba instructor to ensure consistency and correct execution of movements. Each session was organized into three phases and conducted in a group setting. The warm-up lasted 10–15 min and consisted of simple standing rhythmic aerobic movements performed to music with a tempo ranging from 120 to 135 beats per minute (bpm). The main workout lasted 25–45 min and involved choreographed dance routines incorporating standardized Zumba steps [9], performed primarily in a standing position with continuous whole-body movement in response to the instructor’s cues. The complexity of the routines was progressively increased over the course of the intervention, and the music tempo was raised to 140–180 bpm to stimulate cardiovascular fitness and coordination [17]. Dance bouts typically lasted 3–5 min and were interspersed with 15–30 s of active recovery. The cool-down lasted 5–10 min and included lower-intensity standing dance-based movements performed to slower-paced music (~100 bpm).

Training progression was planned across the three intervention phases. During the first four weeks, participants focused on learning basic movements without jumps or complex turns. Between weeks 5 and 8, more elaborate choreographic elements were gradually introduced. During the final phase (weeks 9–12), routines became longer and more demanding, with greater emphasis on coordination, endurance, and overall muscular involvement (Table 1).

Exercise intensity was monitored using Borg’s 6–20 rating of perceived exertion (RPE) scale [18]. This approach was selected because it was practical and feasible for monitoring internal load during supervised group-based field sessions. Session intensity was intended to remain within the ranges of 12–14 (“somewhat hard”) and 15–16 (“hard”), corresponding to moderate-to-vigorous effort [18,19,20]. The mean RPE across the intervention was 14.3 ± 1.3, increasing from 12.2 ± 0.9 in the first session to 15.8 ± 1.1 in the final session, consistent with the intended progression from moderate to vigorous effort. Music selection was standardized and based on official Zumba Fitness choreography materials (ZIN DVDs 48, 50, and Love 75).

### 2.6. Blood Sampling and Analysis

Fasting venous blood samples were collected by venipuncture into heparin-containing tubes between 07:00 and 09:00 a.m. before (pre) and 48 h after the final exercise session of the 12-week intervention. For hematological analyses, blood was collected into K3-EDTA tubes (AFCO, Al-Hanoof Factory for Medical & Lab Supplies, Amman, Jordan) and analyzed using an automated hematology analyzer (Sysmex XN-450 Norderstedt, Germany). ESR was determined from whole blood collected into tubes containing 3.2% sodium citrate using a standard sedimentation pipette (Sediplast; LP Italiana S.p.A., Milan, Italy).

For CRP analysis, blood was collected into heparin-containing tubes. Samples were centrifuged at 1500× *g* for 15 min, and the resulting plasma was analyzed using a CRP latex reagent on an automated biochemistry analyzer (Beckman Coulter AU480, Brea, CA, USA). Samples intended for inflammatory biomarker analysis were stored at −80 °C until analysis, within 3 months of collection. High-sensitivity CRP (hs-CRP) was not measured because it was unavailable in the laboratory at the time of the study. Lipid-related variables were also analyzed within the broader trial protocol but were reported elsewhere [13] and are therefore not detailed in the present manuscript. Detailed information on reagent catalogue numbers, analytical sensitivity, intra- and inter-assay coefficients of variation, duplicate analysis, and specific calibration procedures was not available in the study records and was therefore not reported.

### 2.7. Statistical Analysis

Statistical analyses were performed using SPSS (version 22.0; IBM Corp., Armonk, NY, USA). Data are presented as mean ± standard deviation (SD). Normality was assessed using the Shapiro–Wilk test, and homogeneity of variances was assessed using Levene’s test. Estimated VO_2_max was considered the primary outcome, whereas the hematological and inflammatory outcomes were considered exploratory secondary outcomes. For each continuous outcome, the intervention effect was assessed using analysis of covariance (ANCOVA), with the post-intervention value as the dependent variable, the randomized group as the fixed factor, and the corresponding baseline value as the covariate. Baseline adjustment was applied consistently to all outcomes, irrespective of whether a statistically significant between-group difference was present at baseline. The homogeneity-of-regression-slopes assumption was assessed by testing the group × baseline interaction for each outcome. This assumption was satisfied for all outcomes except PLTs. For PLTs, because the group × baseline interaction was significant, the standard common-slope ANCOVA was not used to infer a single adjusted intervention effect. Instead, an exploratory model retaining the group × baseline PLTs interaction was examined. Given the small sample size and potential sensitivity of this interaction model to heteroscedasticity and leverage, a sensitivity analysis using HC3 heteroscedasticity-robust standard errors was also performed.

No imputation was performed for participants who withdrew before post-testing; therefore, the statistical analyses reflect per-protocol estimates among completers. No formal correction for multiple outcomes was applied. For ANCOVA effects, effect sizes are reported as partial eta squared (ηp^2^) and interpreted as small (0.01 < ηp^2^ < 0.06), medium (0.06 ≤ ηp^2^ < 0.14), and large (ηp^2^ ≥ 0.14) [21]. Adjusted between-group differences are reported with 95% confidence intervals (CIs). Percentage changes (Δ%) from pre- to post-intervention were retained for descriptive purposes. Pre- and post-intervention means and Δ% were used only to describe the direction and magnitude of change; no inferential within-group pre–post tests were used to interpret the intervention effect. Because anthropometric, cardiovascular, and lipid-related outcomes from the same trial have been reported elsewhere [13], the present analyses were restricted to aerobic capacity, hematological parameters, and systemic inflammatory biomarkers. Given the relatively small final sample and the number of exploratory secondary outcomes examined, the exploratory secondary findings were interpreted cautiously, with emphasis on the magnitude and precision of the baseline-adjusted between-group estimates rather than on statistical significance alone. Statistical significance was set at *p* ≤ 0.05.

## 3. Results

**Figure 2:**
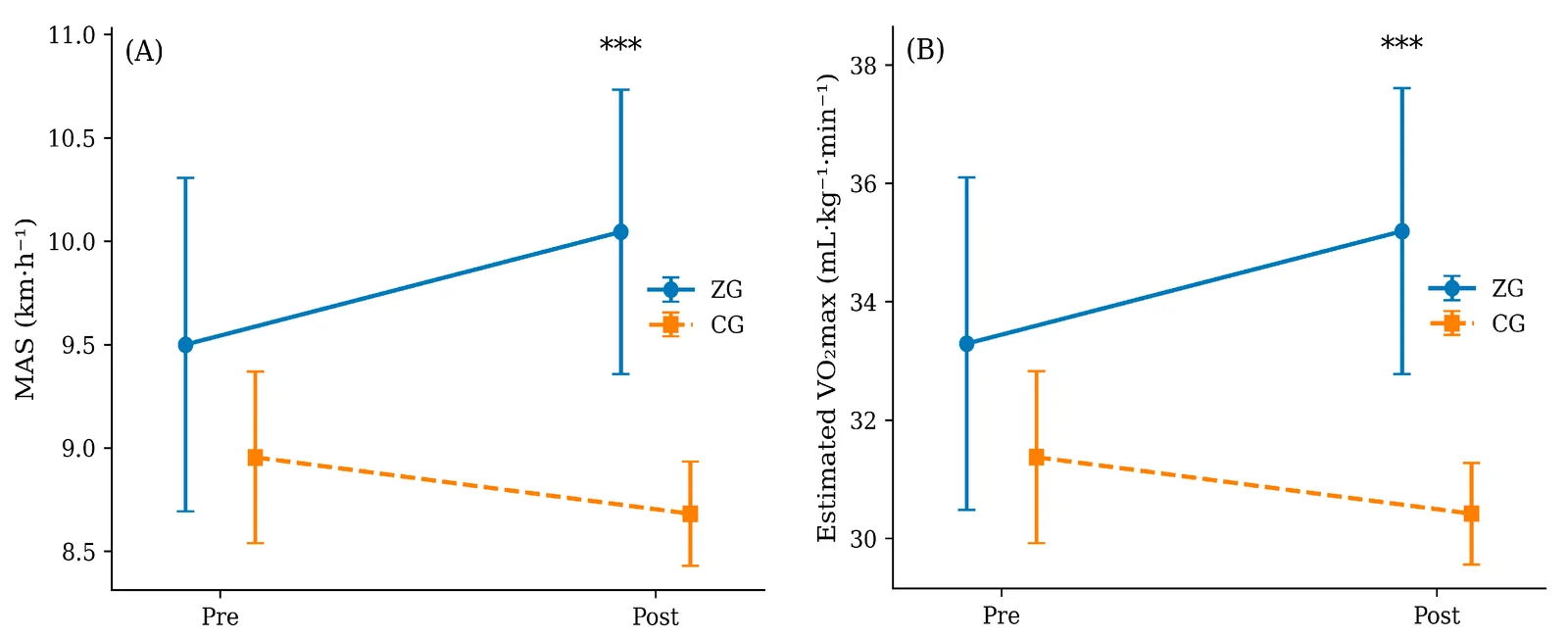
Pre- and post-intervention maximal aerobic speed (MAS) and estimated maximal oxygen uptake (VO_2_max) in the Zumba group (ZG) and control group (CG). (**A**) MAS; (**B**) estimated VO_2_max. Values are presented as mean ± SD. *** Significant baseline-adjusted between-group difference at post-intervention (ANCOVA, *p* < 0.001).

Normality of the data and homogeneity of the variances were confirmed (all *p* > 0.05). Pre- and post-intervention descriptive values for all outcomes are presented in Table 2 and Table 3 and Figure 2. Baseline values were included as covariates in the ANCOVA models for all continuous outcomes, irrespective of the magnitude or statistical significance of between-group differences at baseline. The homogeneity-of-regression-slopes assumption was satisfied for all outcomes except PLTs, for which the group × baseline interaction was significant (*p* = 0.012). Accordingly, the PLTs result was interpreted cautiously.

### 3.1. Aerobic Capacity

Baseline-adjusted ANCOVA showed a significant between-group effect for estimated VO_2_max (F_1,19_ = 40.891, *p* < 0.001, ηp^2^ = 0.683). After adjustment for baseline estimated VO_2_max, the post-intervention value was significantly higher in the ZG than in the CG, with an adjusted between-group difference of 3.59 mL·kg^−1^·min^−1^ (95% CI: 2.41 to 4.76). A corresponding significant baseline-adjusted between-group effect was observed for MAS (F_1,19_ = 41.463, *p* < 0.001, ηp^2^ = 0.686), with an adjusted difference of 1.03 km·h^−1^ (95% CI: 0.69 to 1.36). Descriptively, estimated VO_2_max changed by approximately +6% in the ZG and −2.9% in the CG, whereas MAS changed by approximately +6.1% and −3.0%, respectively (Figure 2).

### 3.2. Hematological Markers

Baseline-adjusted between-group effects were observed for MPV and NEs (Table 2). For MPV, the adjusted group effect was 0.701 fL (95% CI: 0.29 to 1.11; F_1,19_ = 12.668, *p* = 0.002, ηp^2^ = 0.400), with higher adjusted post-intervention values in the ZG. For NEs, the adjusted between-group difference was 0.961 × 10^3^/μL (95% CI: 0.233 to 1.688; F_1,19_ = 7.644, *p* = 0.012, ηp^2^ = 0.287), again with higher adjusted post-intervention values in the ZG.

No significant baseline-adjusted between-group effects were observed for MCV (F_1,19_ = 0.367, *p* = 0.552, ηp^2^ = 0.019), MOs (F_1,19_ = 2.875, *p* = 0.106, ηp^2^ = 0.131), EOs (F_1,19_ = 3.172, *p* = 0.091, ηp^2^ = 0.143), BAs (F_1,19_ = 1.019, *p* = 0.326, ηp^2^ = 0.051), WBCs (F_1,19_ = 3.654, *p* = 0.071, ηp^2^ = 0.161), RBCs (F_1,19_ = 0.499, *p* = 0.489, ηp^2^ = 0.026), HB (F_1,19_ = 0.317, *p* = 0.580, ηp^2^ = 0.016), hematocrit (F_1,19_ = 0.917, *p* = 0.350, ηp^2^ = 0.046), MCH (F_1,19_ = 0.436, *p* = 0.517, ηp^2^ = 0.022), MCHC (F_1,19_ = 3.039, *p* = 0.097, ηp^2^ = 0.138), or LYs (F_1,19_ = 0.063, *p* = 0.805, ηp^2^ = 0.003) (Table 2).

For PLTs, the group × baseline interaction was significant (F_1,18_ = 7.776, *p* = 0.012, ηp^2^ = 0.302). However, this interaction was not confirmed in the HC3 heteroscedasticity-robust sensitivity analysis (interaction coefficient = 0.707, 95% CI: −0.60 to 2.02; *p* = 0.272). Accordingly, the PLTs finding was considered sensitive to the analytical specification, and no robust intervention-specific effect was inferred.

### 3.3. Inflammatory Markers

Baseline-adjusted ANCOVA showed no significant between-group effect for CRP (F_1,19_ = 2.211, *p* = 0.153, ηp^2^ = 0.104) (Table 3). The adjusted post-intervention difference between the ZG and CG was 2.31 mg/L (95% CI: −0.94 to 5.56). Similarly, no significant baseline-adjusted between-group effect was observed for ESR (F_1,19_ < 0.001, *p* = 0.990, ηp^2^ < 0.001) (Table 3). The adjusted ZG-CG difference at post-intervention was 0.08 mm/h (95% CI: −13.16 to 13.32). Accordingly, neither CRP nor ESR showed evidence of an intervention-specific effect after adjustment for the corresponding baseline value.

## 4. Discussion

This randomized pilot trial investigated the effects of a 12-week Zumba training program on aerobic capacity and selected hematological variables and systemic inflammatory biomarkers in overweight/obese postmenopausal women. The principal findings were threefold. First, Zumba training was associated with higher baseline-adjusted post-intervention estimated VO_2_max and MAS compared with the control condition. Second, the hematological response was selective rather than global: baseline-adjusted between-group effects were observed for MPV and NE, whereas no clear intervention-specific effects were identified for the other hematological outcomes. Third, the systemic inflammatory biomarker response was limited, as neither CRP nor ESR showed a significant baseline-adjusted between-group effect.

The finding for estimated VO_2_max represents the clearest outcome of the present study. Exercise training is already well established as an effective strategy for improving cardiorespiratory fitness in postmenopausal women [5,22]. Evidence specific to Zumba is more heterogeneous. Systematic reviews and meta-analyses have reported favorable effects of Zumba on aerobic fitness [8,23,24], and a randomized trial in female employees reported an improvement in VO_2_max following Zumba training [25]. In contrast, a 12-week dance-based Zumba intervention in overweight postmenopausal women did not produce a clear improvement in VO_2_max [26]. Improvements in respiratory function have also been reported following Zumba training in inactive women [9]. Thus, the present findings add to a literature suggesting that cardiorespiratory responses to Zumba may depend on the population, training characteristics, and intervention implementation. However, because VO_2_max was estimated from maximal aerobic speed rather than measured directly by gas exchange, the aerobic findings should be interpreted accordingly. The contribution of the present study is therefore mainly practical rather than mechanistic, suggesting that supervised Zumba may represent one possible aerobic exercise modality for overweight/obese postmenopausal women. Importantly, these findings should not be interpreted as demonstrating a novel physiological effect of exercise on aerobic fitness but rather as extending the evidence concerning Zumba in this specific population.

The hematological findings were more nuanced than the aerobic results and point to selective rather than generalized blood-based adaptations. The clearest baseline-adjusted difference was observed for MPV. MPV reflects platelet size and has been investigated in relation to platelet activity, inflammatory conditions, and cardiovascular phenotypes; however, its biological and clinical interpretation remains complex, and larger PLTs cannot automatically be equated with greater platelet activation or thrombotic risk [27,28,29]. Exercise itself can influence platelet aggregation and function, although the direction and magnitude of these responses appear to depend on exercise modality, intensity, training status, and underlying cardiovascular or metabolic condition [30,31,32]. In the present study, MPV was assessed without direct measures of platelet function. Consequently, because platelet aggregation, soluble P-selectin, thromboxane B_2_, or other functional markers were not assessed, the MPV finding should be considered exploratory and should not be interpreted as direct evidence of either a pro-thrombotic or anti-thrombotic adaptation.

Interpretation of PLTs count itself also requires caution. The assumption of homogeneous regression slopes was not satisfied, and although an exploratory model suggested that the baseline-post-intervention relationship differed between groups, this finding was not confirmed by the robust sensitivity analysis. Thus, the PLTs result was sensitive to analytical specification and does not provide robust evidence of an intervention-specific effect.

NEs represented the second hematological variable showing a baseline-adjusted between-group difference. NEs are highly responsive to exercise and neuroendocrine regulation, and their circulating concentrations may vary according to exercise exposure, intensity, adiposity, inflammatory status, and participant characteristics [33,34,35,36]. Catecholamines and glucocorticoids are important regulators of leukocyte trafficking and can induce redistribution of circulating neutrophils [35,36]. However, blood sampling in the present study was performed 48 h after the final exercise session, making an immediate exercise-induced leukocyte redistribution an unlikely sole explanation for the observed difference. Of particular relevance, aerobic exercise has previously been shown to modify WBCs and neutrophil profiles in overweight/obese postmenopausal women, with responses influenced by exercise dose and baseline inflammatory status [33]. Exercise-related immune responses may also vary according to the characteristics of the training stimulus [34,37]. Nevertheless, because NEs are nonspecific immune-related hematological indices rather than direct markers of systemic inflammation, the present finding should be interpreted as an exploratory leukocyte-profile response rather than evidence of an anti-inflammatory or pro-inflammatory effect.

No clear baseline-adjusted intervention effects were observed for the remaining hematological variables. Therefore, the descriptive pre–post variations in these outcomes should not be interpreted as training-induced adaptations. Given the small sample size and the use of single pre- and post-intervention blood measurements, these hematological findings may also be influenced by biological, analytical, or pre-analytical variability. The blood-cell findings should therefore be viewed as preliminary and hypothesis-generating rather than evidence of a generalized hematological adaptation to Zumba.

The systemic inflammatory biomarker findings do not support a clear intervention-specific anti-inflammatory effect as assessed by CRP and ESR. This interpretation does not conflict with the NE finding because leukocyte subfractions and systemic inflammatory biomarkers represent different biological domains. After baseline adjustment, neither CRP nor ESR differed clearly between the intervention and control conditions. Previous evidence in overweight/obese postmenopausal women indicates that exercise can influence inflammatory biomarkers, but responses vary substantially across markers and exercise modalities [10]. For example, reductions in CRP have been reported after longer-term aerobic exercise [38] and after a 12-week resistance-training intervention [39]. These findings suggest that the inflammatory response cannot be attributed simply to intervention duration; exercise modality, training dose, baseline inflammatory status, and the biomarker examined are also likely to be important. The use of standard CRP rather than hs-CRP, together with the small pilot sample, may additionally have limited the ability to detect modest changes in low-grade systemic inflammation. Overall, the present results provide no evidence of a robust systemic anti-inflammatory response to the 12-week Zumba intervention as assessed by CRP and ESR.

From an applied perspective, a supervised 12-week Zumba program may represent a feasible aerobic exercise option for overweight/obese postmenopausal women. Because Zumba combines structured aerobic activity with a group-based dance format, it may offer an alternative to more conventional exercise modalities [8,26]. However, the present study did not directly assess enjoyment, adherence determinants, or long-term exercise participation; therefore, potential behavioral advantages of Zumba should not be inferred from the current data. The hematological findings, particularly those involving MPV and NE, require substantially greater caution than the primary aerobic outcome because of the pilot sample size, multiple exploratory outcomes examined, and absence of formal multiplicity correction. Future studies should confirm these findings in adequately powered randomized trials incorporating longer follow-up, direct measurement of aerobic capacity, intention-to-treat analyses, and more comprehensive assessment of PLT function, leukocyte activity, and systemic inflammatory biomarkers.

Several limitations should be acknowledged. The final sample size was relatively small, which limits statistical power and generalizability, particularly for biologically variable hematological and inflammatory outcomes. In addition, 8 of the 30 randomized participants (26.7%) withdrew before the post-intervention assessment, and the analyses were restricted to study completers. Although baseline assessments were performed before randomization, the individual-level baseline raw data for participants who subsequently withdrew could not be retrieved from the archived dataset available for this secondary analysis. Consequently, completers and non-completers could not be compared at baseline, potential attrition bias could not be directly evaluated, and an intention-to-treat analysis could not be performed. The findings should therefore be interpreted as effects among participants who completed the study rather than definitive effects among all randomized participants. For PLT, interpretation was additionally limited by violation of the homogeneity-of-regression-slopes assumption and by the lack of confirmation of the interaction in the robust sensitivity analysis. Given the pilot sample size and the number of exploratory secondary outcomes examined, the hematological and inflammatory findings should be interpreted cautiously. Furthermore, no formal adjustment for multiple outcomes was applied; therefore, the possibility of type I error cannot be excluded, particularly for the exploratory hematological findings. Dietary intake and habitual physical activity outside the intervention were not objectively monitored and may have influenced blood-based outcomes. VO_2_max was estimated from MAS rather than directly measured through respiratory gas exchange, and the selected field protocol requires further validation specifically in overweight/obese postmenopausal women. Systemic inflammation was assessed only using standard CRP and ESR, while leukocyte subfractions such as NE and MO are nonspecific hematological indices rather than direct measures of inflammatory activity. The absence of hs-CRP, cytokines, leukocyte activation markers, and functional immune assays limits mechanistic interpretation. Similarly, the use of single pre- and post-intervention blood samples may not adequately capture biological variability. Training intensity was monitored primarily using RPE, which is practical in applied group-based settings but subjective, and the absence of objective heart-rate monitoring limits the precision of exercise-intensity quantification. Finally, the study included women recruited from a single geographical region, which may limit the external validity of the findings.

## 5. Conclusions

In conclusion, 12 weeks of Zumba training (3 sessions/week) were associated with higher baseline-adjusted post-intervention estimated VO_2_max and MAS in overweight/obese postmenopausal women compared with the control condition. The intervention was also associated with selective baseline-adjusted between-group differences in hematological variables, particularly MPV and NE, whereas no clear intervention-specific effects were identified for the remaining hematological outcomes. Given the pilot sample, multiple secondary outcomes examined, and absence of formal multiplicity correction, the MPV and NE findings should be regarded as exploratory and hypothesis-generating. CRP and ESR did not show significant baseline-adjusted between-group effects, indicating that the 12-week Zumba program did not produce a robust systemic anti-inflammatory effect detectable with these markers. These pilot findings suggest that supervised Zumba may be a feasible aerobic exercise option, adding to the limited evidence available for this specific exercise modality in this population.

## Figures and Tables

**Table 1:** Zumba training program.

Weeks	1–4	5–8	9–12
Frequency	3×/Week	3×/Week	3×/Week
Warm-up	Rhythmic movements to music (120–135 bpm): step touch, double step touch, side-to-side, leg curl, double leg curl, knee lift, double knee lift, V-step, squat, hop.	Rhythmic movements to music (120–135 bpm): step touch, double step touch, side-to-side, leg curl, double leg curl, knee lift, double knee lift, V-step, squat, hop.	Rhythmic movements to music (120–135 bpm): step touch, double step touch, side-to-side, leg curl, double leg curl, knee lift, double knee lift, V-step, squat, hop.
Duration	15 min	15 min	10 min
Main Choreography Phase	Basic Zumba steps without rotations or jumps. Simple choreographies (step, salsa, mambo, tango, turns, grapevine, cha-cha-cha) to music (140–180 bpm). 3–5 min segments, 15–30 s rest.	More complex choreographies, gradual introduction of new steps, routines from tracks 48 and 50 of ZIN DVD to music (140–180 bpm). Engages thighs, glutes, abs, arms.	Advanced choreographies, longer sequences, highest complexity. Maintains 3–5 min dance/rest intervals. Routines from tracks 48, 50, and Love (ZIN75 DVD) to music (140–180 bpm).
Duration	25 min	35 min	45 min
Cool-down	Gentle dance movements to calming music (100 bpm).	Gentle dance movements to calming music (100 bpm).	Gentle dance movements to calming music (100 bpm).
Duration	10 min	10 min	5 min

**Table 2:** Changes in hematological biomarkers from pre- to post-test following the 12-week intervention in the Zumba group (ZG) and the control group (CG).

Variables	Group	Pre	Post	Δ%
WBCs (10^3^/μL)	ZG	6.2 ± 1.7	6.15 ± 1.7	−0.1
	CG	5.5 ± 1.0	4.8 ± 1.2	−14.1
RBCs (10^6^/μL)	ZG	4.8 ± 0.4	4.8 ± 0.3	−0.4
	CG	4.8 ± 0.3	4.9 ± 0.8	+2.9
HB (g/dL)	ZG	12.9 ± 0.9	13.0 ± 0.9	+0.8
	CG	13.0 ± 0.6	13.3 ± 1.7	+2.9
Hematocrit (%)	ZG	41.0 ± 2.5	41.3 ± 2.4	+0.5
	CG	41.6 ± 2.2	43.0 ± 5.6	+4.0
MCV (fL)	ZG	86.0 ± 3.9	86.7 ± 3.8	+0.9
	CG	86.7 ± 4.4	87.8 ± 3.9	+1.2
MCH (pg)	ZG	26.98 ± 1.35	27.28 ± 1.53	+1.1
	CG	27.03 ± 1.55	27.09 ± 1.28	+0.2
MCHC (g/dL)	ZG	31.39 ± 0.67	31.49 ± 0.94	+0.3
	CG	31.19 ± 0.58	30.87 ± 0.48	−1.0
MPV (fL)	ZG	8.15 ± 0.57	8.57 ± 0.66	+5.2
	CG	8.10 ± 1.12	7.84 ± 0.56	−3.1
PLTs (10^3^/μL)	ZG	258.54 ± 82.99	240.18 ± 65.80	−7.1
	CG	235.36 ± 64.11	237.31 ± 51.89	+0.8
NEs (10^3^ /μL)	ZG	2.79 ± 0.89	3.12 ± 1.05	+11.7
	CG	3.00 ± 0.82	2.27 ± 0.73	−24.4
LYs (10^3^/μL)	ZG	2.59 ± 0.87	2.46 ± 0.53	−5.3
	CG	2.04 ± 0.35	2.15 ± 0.38	+5.6
MOs (10^3^/μL)	ZG	0.53 ± 0.15	0.27 ± 0.26	−48.6
	CG	0.41 ± 0.08	0.04 ± 0.02	−88.5
EOs (10^3^/μL)	ZG	0.22 ± 0.14	0.07 ± 0.05	−69.9
	CG	0.12 ± 0.06	0.03 ± 0.02	−74.1
BAs (10^3^/μL)	ZG	0.04 ± 0.02	0.03 ± 0.02	−25.0
	CG	0.017 ± 0.010	0.016 ± 0.008	−5.3

Note. Values are expressed as mean ± SD; Δ%: percentage change. Positive values indicate an increase, and negative values indicate a decrease. WBCs: white blood cells; RBCs: red blood cells; HB: hemoglobin; MCV: mean corpuscular volume; MCH: mean corpuscular hemoglobin; MCHC: mean corpuscular hemoglobin concentration; PLTs: platelets; MPV: mean platelet volume; NEs: neutrophils; LYs: lymphocytes; MOs: monocytes; EOs: eosinophils; BAs: basophils.

**Table 3:** Changes in inflammatory biomarkers from pre- to post-test following the 12-week intervention in the Zumba group (ZG) and the control group (CG).

Variables	Group	Pre	Post	Δ%
CRP (mg/L)	ZG	4.50 ± 4.55	7.01 ± 5.21	+55.4
	CG	1.92 ± 1.85	2.88 ± 2.57	+49.5
ESR (mm/h)	ZG	23.4 ± 15.4	27.2 ± 19.1	+16.3
	CG	10.7 ± 6.6	18.0 ± 10.0	+67.8

Note. Values are expressed as mean ± SD; Δ%: percentage change. Positive values indicate an increase, and negative values indicate a decrease. CRP: C-reactive protein; ESR: erythrocyte sedimentation rate.

## Data Availability

The data that support the findings of this study are available from the corresponding author upon reasonable request, subject to ethical and privacy restrictions.

## Abbreviations

ANCOVA	Analysis of covariance
ANOVA	Analysis of variance
BAs	Basophils
BF	Body fat percentage
bpm	Beats per minute
CG	Control group
CI	Confidence interval
CRP	C-reactive protein
EOs	Eosinophils
ESR	Erythrocyte sedimentation rate
HB	Hemoglobin
hs-CRP	High-sensitivity C-reactive protein
K3-EDTA	Tripotassium ethylenediaminetetraacetic acid
LYs	Lymphocytes
MAS	Maximal aerobic speed
MCH	Mean corpuscular hemoglobin
MCHC	Mean corpuscular hemoglobin concentration
MCV	Mean corpuscular volume
MOs	Monocytes
MPV	Mean platelet volume
NEs	Neutrophils
PLTs	Platelets
RBCs	Red blood cells
RPE	Rating of perceived exertion
SD	Standard deviation
VO_2_max	Estimated maximal oxygen uptake
WBCs	White blood cells
ZG	Zumba group
